# Correlation of NF-κB signal pathway with tumor metastasis of human head and neck squamous cell carcinoma

**DOI:** 10.1186/1471-2407-10-437

**Published:** 2010-08-17

**Authors:** Ming Yan, Qin Xu, Ping Zhang, Xiao-jian Zhou, Zhi-yuan Zhang, Wan-tao Chen

**Affiliations:** 1 Department of Oral and Maxillofacial Surgery, Ninth People's Hospital, Shanghai Jiao Tong University School of Medicine, Shanghai 200011, China; 2 Shanghai Key Laboratory of Stomatology, Shanghai 200011, China

## Abstract

**Background:**

Nuclear factor-kappa B (NF-κB) signaling constitutes a key event in the multistep process of carcinogenesis, progression and treatment in many cancer types. However, the significance of NF-κB pathway for complex and tissue-specific aspects of head and neck cancer progression, such as invasion and metastasis, is less understood.

**Methods:**

The expression of NF-κB p65 in squamous cell carcinoma of the head and neck (SCCHN) clinical specimens by immunohistochemistry. The role of NF-κB activity in head and neck squamous cell carcinoma was determined by western blot, reporter assay and EMSA analysis *in vitro *and metastasis assays *in vivo *in different metastatic potential tumor cells. Furthermore, the apoptosis rate and expression of metastasis-related protein such as MMP9 and VEGF were examined by Annexin V/PI staining and Western blot, respectively.

**Results:**

A higher level of active nuclear-localized NF-κB was observed in the metastatic SCCHN specimens group (*p *< 0.01). The NF-κB activities of SCCHN cell lines with different metastatic potentials were then determined and in excellent agreement with results found in SCCHN specimens, highly metastatic SCCHN cell lines expressed high level of NF-κB activity. The treatment of highly metastatic SCCHN cells with NF-κB inhibitors reduced the *in vitro *cell invasion capacity of the cells without affecting the apoptotic rate. Additionally, the NF-κB inhibitors significantly inhibited the experimental lung metastasis of Tb cells and lymph node metastasis of TL cells in nude mice. Furthermore, the expression of metastasis-related proteins, such as matrix metalloproteinase 9 and vascular endothelial growth factor, was inhibited by pyrrolidine dithiocarbonate.

**Conclusions:**

This study suggests that NF-κB activity significantly contributes to tumor hematologic and lymphatic metastases and may aid in the development of early detection methods or therapies targeting non-conventional molecular targets.

## Background

Head and neck cancer (HNC) consistently ranks as one of the most prevalent cancers worldwide. Over 90% of all HNC are squamous cell carcinomas [1]. Worldwide, more than 650,000 new cases with HNC are diagnosed every year [2], and two-thirds of patients with HNC present with locally advanced lesions and/or regional lymph node involvement. In the United States, 35,720 new cases of oral cavity & pharynx cancer were diagnosed in 2009[3]. Curative surgery, radiotherapy and chemotherapy have failed to reduce the overall mortality rate of head and neck cancers over the past several decades. Therefore, it is absolutely necessary to determine the mechanisms contributing to invasion and metastasis of squamous cell carcinoma of the head and neck (SCCHN).

Recently, accumulating evidence has suggested that the nuclear factor-κB (NF-κB) signaling pathway plays a critical role in carcinogenesis, protection from apoptosis and chemoresistance in a number of cancer types, including head and neck cancer, breast cancer, hepatocellular carcinoma and gastric cancer [3-7]. NF-κB is a transcription factor that is retained in the cytoplasm by the inhibitory protein IκB. Phosphorylated IκBα is ubiquitinated and subsequently degraded by the 26S proteasome, resulting in the liberation of NF-κB. NF-κB can then enter the nucleus to regulate the expression of genes involved in cell proliferation, cell survival and apoptosis [8,9]. Several studies have suggested that NF-κB is also associated with cancer cell invasion and metastasis [5,10-13]. However, the presence and role of NF-κB in the invasion and metastasis of cancer is not clear.

The present study provides evidence that NF-κB activity significantly contributes to tumor invasion and metastasis. The data presented here demonstrates that inhibition of the NF-κB signaling pathway could reduce tumor invasion and metastasis *in vitro *and *in vivo*. Therefore, NF-κB, as well as its downstream or upstream signaling effectors, may be effective molecular targets for the detection or inhibition of SCCHN hematologic and lymphatic metastasis.

## Methods

### Immunohistochemistry

This study was approved by the Institutional Review Board of the Ninth People's Hospital, Shanghai Jiao Tong University School of Medicine. All specimens for this study were obtained from surgical samples with pathological diagnosis and informed consent. The paraffin-embedded tissue blocks were selected based on patient diagnosis: primary tumors without lymph node involvement (Tn), primary tumors with lymph node involvement (Tm) and the tissues of their paired metastatic lymph node (Lm). There were a total of 30 specimens in each group (Table 1). Patients who were previously treated (radiotherapy or chemotherapy) for the index tumor or another head and neck primary tumor within the past five years were excluded. The tumor size, nodal metastases and distant metastases (TNM) classification of all tumors was according to the International Union Against Cancer. The clinical information was obtained from the surgical pathology files in the hospital. The relevant clinical parameters, including age, gender, alcohol use, smoking habits, original primary tumor site and pathology stage, are listed in Table 2.

**Table 1:** Clinical characteristics of patients with head and neck squamous cell carcinoma

Patients (Tn)	Age/gender	Tumor site	Pathological stage	Patients (Tm/Lm)	Age/gender	Tumor site	Pathological stage
No.				No.			
1	48/M	Buccal mucosa	T4N0M0	1	35/F	Tongue	T2N1M0
2	49/F	Tongue	T3N0M0	2	42/M	Mandibular alveolar	T2N1M0
3	60/F	Buccal mucosa	T2N0M0	3	59/M	Buccal mucosa	T4N2bM0
4	35/M	Upper lip	T1N0M0	4	62/F	Mandibular gingival	T4N2bM1
5	64/F	Tongue	T2N0M0	5	63/F	Tongue	T2N1M0
6	61/M	Soft palate and buccal mucosa	T1N0M0	6	53/M	Buccal mucosa	T2N2aM0
7	73/M	Lower lip	T1N0M0	7	72/F	Tongue	T1N2aM0
8	81/M	Lower lip	T1N0M0	8	65/F	Tongue	T2N2bM0
9	69/F	Lower lip	T2N0M0	9	50/M	Tongue	T2N1M0
10	55/M	Tongue	T1N0M0	10	72/M	Mandibular gingival	T4N1M0
11	76/M	Buccal mucosa	T2N0M0	11	41/M	Tongue	T2N1M0
12	56/F	Mandibular gingiva	T1N0M0	12	48/M	Tongue and floor of mouth	T3N2bM0
13	68/F	Buccal mucosa	T2N0M0	13	70/M	Tongue	T3N1MO
14	42/M	Tongue	T2N0M0	14	74/M	Soft palate	T4N1M0
15	73M	Tongue	T1N0M0	15	45/M	Tongue	T4N2bM0
16	42/F	Tongue	T2N0M0	16	62/M	Tongue	T2N1M0
17	52/F	Tongue	T2N0M0	17	56/M	Floor of mouth	T4N2cM0
18	50/M	Tongue	T1N0M0	18	55/M	Tongue	T4N2M0
19	51/F	Mandibular gingival	T3N0M0	19	51/M	Buccal mucosa	T3N2aM0
20	51/M	Buccal mucosa	T3N0M0	20	58/F	Tongue	T2N1M0
21	63/F	Tongue	T2N0M0	21	51/M	Buccal mucosa	T1N1M0
22	55/M	Tongue	T2N0M0	22	50/M	Glottis	T2N3M0
23	62/F	Tongue	T2N0M0	23	49/M	Tongue	T2N2M0
24	58/F	Buccal mucosa	T4N0M0	24	75/M	Tongue	T4N1M0
25	79/M	Floor of mouth	T4N0M0	25	42/F	Tongue and floor of mouth	T4N1M0
26	52/M	Buccal mocosa	T2N0M0	26	68/F	Buccal mucosa	T4N2aM0
27	78/F	Mandibular alveolar	T2N0M0	27	62/M	Tongue	T3N2bM0
28	30/M	Tongue	T3N0M0	28	62/F	Mandibular gingival	T2N2M0
29	42/F	Mandibular gingival	T1N0M0	29	36/M	Tongue	T4N2aM0
30	56/M	Hard palate	T1N0M0	30	50/M	Tongue	T4N1M0

**Table 2:** Clinical correlation

Characteristics	Tn	Tm/Lm	df	χ^2^	P-value
Age(year)*					0.5776
Mean	57.7	55.9			
Std Dev	13.1	11.3			
Sex			1	1.763	0.184
Male	16	21			
Female	14	9			
Smoking habit			1	2.052	0.152
Yes	6	11			
No	24	19			
Alcohol use			1	0.287	0.592
Yes	10	12			
No	20	18			
Tumor site			2	2.400	0.121
Tongue	12	18			
Other sites	18	12			
Differentiation			1	17.778	0.001
WD	20	4			
MOD/PD	10	26			

Abbreviations: PD, poor differentiated; WD, well differentiated; MOD, moderate differentiated.

*P value was obtained using grouped *t *test, others were obtained using χ^2 ^or Wilcoxon Rank-sum Test for comparison between two groups or more than two groups, respectively.

NF-κB p65 expression in the tissue specimens was determined using a mouse monoclonal anti-human NF-κB p65 antibody (Sigma-Aldrich). Mouse IgG at a 1:100 dilution was used as a negative control. Immunohistochemical analysis of formalin-fixed, paraffin-embedded human specimens was performed according to a modified procedure. Briefly, after deparaffinization with xylene and rehydration with ethanol, endogenous peroxidase activity was blocked by incubating the slides in 3% hydrogen peroxide in methanol for 15 minutes. To retrieve the antigens, the tissue slides were heated in a microwave oven in 100 mM sodium citrate buffer (pH 6.0) for 10 minutes and then cooled to room temperature for 20 minutes. Cover tissue sections with 3-4 drops of protein block buffer (DAKO) and incubate slides for 15 minutes in humid chamber at room temperature. After being washed in phosphate-buffered saline (PBS), the slides were incubated with a 1:50 dilution of the anti-NF-κB p65 antibody overnight at 4°C. The next day, the slides were washed with PBS and incubated with a horseradish peroxidase (HRP)-conjugated affinity purified goat anti-mouse IgG (H+L) secondary antibody for one hour at room temperature and with a biotin-avidin peroxidase conjugate (ABC kit, Vector Laboratories) for 15 minutes at room temperature. The substrate (0.1% 3,3 - diaminobenzidine solution; Sigma, St. Louis, MO) was then added in PBS with 0.01% hydrogen peroxide. Finally, the slides were counterstained with hematoxylin (Vector Laboratories) for 50 seconds and then observed using light microscopy. The immunoreactivity of the tissue was estimated by counting the number of nuclear staining-positive cells per 1 000 tumor cells. Independent counts were made by three pathologists with no prior knowledge of the clinical information. The specimens were divided into five groups: > 80% nuclear staining-positive cells, 50%-80% nuclear staining-positive cells, 30%-49% nuclear staining-positive cells, 10%-29% nuclear staining-positive cells and < 10% nuclear staining-positive cells.

### Cell culture

Tca8113, a human SCCHN cell line, was derived from a patient with a tongue squamous cell carcinoma. The Tb cell line was selected from the Tca8113 cell line using a combination of selection *in vivo *and clone selection *in vitro*. Compared to non-metastatic Tca8113, the Tb cell line demonstrated an increased (up to 100%) metastasis rate in an experimental lung metastasis model using nude mice. In contrast, TL cells, another derivative of the Tca8113 cell line, possess high metastatic potential to regional lymph nodes. The TSCC cell line is a non-metastatic cell line established at Wuhan University. The OSC-4 cell line has a strong invasion tendency and was developed at Kochi University, Japan. All five of these cell lines were maintained in RPMI 1640 with 10% fetal bovine serum (FBS) supplemented with 1 mM L-glutamine, 100 U/ml penicillin and 100 U/ml streptomycin and were kept at 37°C and 5% CO_2_.

### Western blot

Cells were treated with 50 ng/mL tumor necrosis factor-α (TNFα; Cytolab, Peprotech ASIA) for the indicated time. The cells were then harvested and washed twice with PBS. The cells were pelleted and lysed with lysis buffer containing 1% Nonidet P-40, 5% sodium deoxycholate, 1 mM PMSF, 100 mM sodium orthovanadate and 1% protease inhibitor cocktail (Sigma-Aldrich, USA). The protein concentration was determined using the bicinchoninic acid (BCA) Protein Assay Kit according to the manufacturer's protocol (Pierce), and the cell extracts were stored at -80°C until use. Before loading, the protein samples were boiled in sample buffer [62.5 mM Tris-HCl (pH 6.8), 10% (w/v) glycerol, 100 mM DTT, 2.3% SDS, 0.002% bromophenol blue] for 10 minutes. The samples were separated in a 10% SDS-PAGE gel and transferred to a polyvinylidene difluoride membrane (Amersham Pharmacia Biotech, Arlington Heights, IL). The membranes were blotted with 5% fat-free milk overnight at 4°C and probed with the primary antibodies. The immunocomplexes were visualized using an HRP-conjugated goat anti-rabbit or anti-mouse antibody (Promega) and ECL plus reagents (Amersham Pharmacia Biotech, Arlington Heights, IL). The anti-IκBα, anti-phospho-IκBα (serine 32) and anti-phospho-NF-κB p65 (serine 536) polyclonal antibodies were from Cell Signaling Technology, while the anti-MMP9 and anti-VEGF antibodies were from Santa Cruz Biotech (CA, USA).

### NF-κB luciferase reporter assay

The SCCHN cells (2×10^5^) were plated in a 6-well dish and transfected with the 2× NF-κB-luciferase plasmid using Lipofectamine 2 000 according to the manufacturer's instructions (Invitrogen Life Technologies). The cells were co-transfected with the pPenilla Renilla luciferase reporter to normalize for transfection efficiency. Six hours after transfection, the transfection medium was replaced with growth medium, and the cells were incubated at 37°C for an additional 48 hours. A second group of cells was treated with TNFα (50 ng/ml) for 12 hours, and the luciferase activity was measured using the Dual Luciferase System (Promega, USA).

### Electromobility Shift Assay (EMSA)

DNA-binding assays were carried out using nuclear extracts isolated from cells in the log phase of growth. The nuclear extracts were isolated using NE-PER Nuclear and Cytoplasmic Extraction Reagents (Pierce, USA). Synthetic complementary oligonucleotides were 3'-biotinylated using a Biotin 3'-End DNA Labeling Kit (Pierce) according to the manufacturer's instructions and annealed for two hours at room temperature. The sequence of the oligonucleotides used is: 5'-AGTTGAGGGGACTTTCCCAGGC-3' and 3'-TCAACTCCCCTGAAAGGGTCCG-5', which contains a putative binding site for wild-type NF-κB. Meanwhile, the other sequence of the oligonucleotides used is: 5'-AGTTGAGGCGACCTTTAAAAGGC-3' and 3'-TCAACTCCCCTGAAAGGGTCCG-5', which contains a mutant NF-κB binding site as the underline shown. The binding reactions were carried out for 20 minutes at room temperature in the presence of 50 ng/μL poly(dI-dC), 0.05% Nonidet P-40, 5 mM MgCl_2_, 10 mM EDTA and 2.5% glycerol in 1× binding buffer (LightShift Chemiluminescent EMSA Kit, Pierce) using 20 fmol of the biotin- labeled target DNA and 5 μg of the nuclear extract. As controls, lane 1 contained only the biotin-end-labeled probe and lane 2 contained the labeled mutant probe only. For the unlabeled and mutant probe competition, extracts were pre-incubated with a 100-fold excess of the unlabeled probe before the addition of the labeled probe (lane 3 and lane 4). Both the biotin-end-labeled probe and the nuclear extracts were added to lane 10 as a positive control. Reactions were loaded into a native 4% polyacrylamide gel (pre-ran for 60 minutes) in 0.5 × Tris borate/EDTA, ran at 100 V and transferred onto a positively charged nylon membrane (Hybond-N^+^) in 0.5 × Tris borate/EDTA at 100 V for 30 minutes. The transferred DNA was immediately cross-linked to the membrane for 15 minutes on a UV transilluminator equipped with 312 nm bulbs and detected using HRP-conjugated streptavidin (LightShift Chemiluminescent EMSA Kit) according to the manufacturer's instructions.

### Immunofluorescence

Cells were stimulated with either 50 ng/mL TNFα for 30 minutes, 100 μM pyrrolidine dithiocarbonate (PDTC) or 10 μM BAY 11-7085 for 1 hour. Two additional groups were treated with PDTC or BAY 11-7085 for 30 minutes and then TNFα was added for an additional 30 minutes. After incubation, the cells were fixed with 4% formaldehyde and permeabilized with 0.1% Triton X-100. After blocking in 3% BSA for 30 minutes, the cells were incubated for one hour with an anti-human p65 antibody (Santa Cruz, clone F-6, dilution 1:100), then washed and incubated for 30 minutes with an Alexa Fluor 488-conjugated anti-mouse IgG F(ab')_2 _fragment (Invitrogen Molecular Probe, dilution 1:200) at room temperature in the dark. The cells were washed three times with PBS containing 0.02% Tween 20 and mounted in aqueous mounting medium containing 0.5 mg/ml 40-6-diamidino-2-phenylindole to stain the nuclei. Three random visual fields at a 200 × magnification were observed using a Leica TCS SP2 confocal spectral microscope.

### Cell migration assays

The effect of NF-κB activation on cell migration was examined using Matrigel Invasion Chambers, as suggested by the manufacturer (BD Bioscience). The upper surface of the chamber contained the transwell filter (8 μm pores) coated with Matrigel, fibronectin and vitronectin. Cells (1×10^5^) were collected and placed in the upper chamber with 50 ng/ml TNFα, 100 μM PDTC or 10 μM BAY 11-7085. Treated cells with the vehicles of above reagents were used as a control. The cells were incubated for 24 hours in a humidified tissue culture incubator at 37°C and 5% CO_2_. The next day, a cotton-tipped swab was used to remove the non-invading cells by applying gentle but firm pressure while moving the tip around the membrane surface. The cells on the lower surface of the insert were stained with hematoxylin for 10 minutes. The number of invading cells was counted using a microscope at a 400× magnification, and the mean number of cells per field in five random fields was recorded. The extent of cell invasion is expressed as the fold increase in the total number of cells on the lower surface of the chamber in the treated samples compared to the total number of invading cells in the untreated samples.

### Mice Injected with Tb Cells and PDTC Treatment

Female 4- to 5-week-old nu/nu Balb/C mice (18-20 g) were maintained in a specific pathogen-free animal facility of the Laboratory of Animal Experiment Affilited to Ninth People's Hospital, Shanghai Jiao Tong University School of Medicine. There were 10 mice in each group. Logarithmically growing Tb cells were harvested and injected into the tail vein of the mice at a dose of 2×10^6 ^cells per mouse. The mice were injected intraperitoneally (i.p) with PDTC (Sigma-Aldrich) at a concentration of 1 mM/kg/day in 100 μl of PBS for 14 days beginning on the day of the injection of the Tb cells. Control animals received 100 μl of PBS alone. The mice were checked daily for weight loss and trouble breathing as a sign of massive lung metastases. All the mice were euthanized when the control mice lost 30% of their body weight due to lung metastasis (an average of six weeks after the tail vein injection). The lungs were excised and collected in PBS, and the lung weight was determined for each individual animal. For histological analysis of metastasis, the lungs were immersed in 10% neutral buffered formalin before paraffin embedding and sectioning. Sections (5 μm thick) were stained using hematoxylin and eosin (H&E) and evaluated. The total number of metastases per lung was determined by collection of serial lung sections, selection of sections approximately 0.3 mm apart and counting the number of metastatic lesions. Large metastatic lesions that appeared in more than one section were only counted once.

### Mice Injected with TL Cells and PDTC or BAY 11-7085 Treatment

Female 4- to 5-week-old Balb/C^nu/nu ^mice were maintained as described above. The mice were divided into three groups: a control group, a PDTC group and a BAY 11-7085 group, with five mice in each group. Logarithmically growing TL cells were harvested and injected into the foot pads of the mouse at a dose of 2×10^6 ^cells for each foot pad. Three weeks after injection, the mice were injected intraperitoneally (i.p) with either 50 mM/kg/day of PDTC (Sigma-Aldrich) or 5 mM/kg/day of BAY 11-7085 (Calbiocham) in 100 μl PBS for 14 days. Control animals received 100 μl PBS alone. The mice were checked daily for weight loss and popliteal fossa lymph node metastasis. All the mice were euthanized an average of nine weeks after injection. For histological analysis of metastasis, the popliteal fossa lymph nodes were excised and immersed in 10% neutral-buffered formalin before paraffin embedding and sectioning. Sections (5 μm thick) were processed for H&E staining and histological evaluation. The number of lymph nodes with metastases was determined by three pathologists.

### Annexin V/PI staining

Tca8113 and TL cells (1×10^6^) treated with TNFα (50 ng/ml), PDTC 100 μM and BAY 11-7085(10 μM) alone or with TNFα plus PDTC or plus BAY 11-7085 for 24 hours were labeled with FITC-conjugated Annexin V and propidium iodide (PI) using the Annexin V-FITC/PI Apoptosis Detection Kit (BD Pharmingen) according to the manufacturer's protocol. The stained cells were analyzed using flow cytometry (FACScalibur, Becton Dickinson, Rutherford, NJ) to distinguish between viable (Annexin V-/PI-), early apoptotic (Annexin V+/PI-), late apoptotic (Annexin V+/PI+) or dead (Annexin V-/PI+) cells. The apoptotic rate was calculated as the percentage of early apoptotic cells plus the percentage of late apoptotic cells.

## Results

### Expression of NF-κB p65 in Primary SCCHN Tumors and Lymph Node Metastases

**Figure 1:**
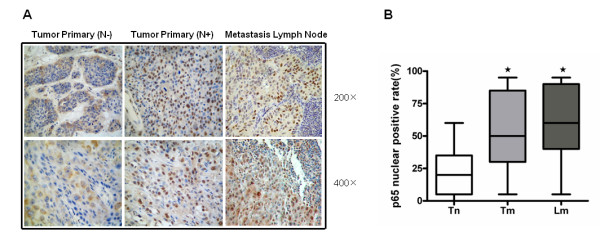
**Immunologic detection of NF-κB p65 in SCCHN tissues**. (a) Representative images of p65 immunostaining of a primary tumor with lymph node involvement (N+) (Tm), the corresponding lymph node contain metastases (Ln) from the same patient and a primary tumor without lymph node involvement (N-) (Tn). (b) Graphical representation of the percentage of p65-positive nuclei in the SCCHN tissue. The asterisk (*) denotes *p *< 0.01.

The expression of NF-κB p65 in primary SCCHN tumors and lymph node metastases was examined using immunohistochemistry. The clinical parameters of the patients are shown in Table 1. The tissue samples included 30 primary tumors with lymph node involvement (Tm), along with their lymph node contain metastases (Lm), and 30 primary tumors without lymph node involvement (Tn) specimens. No significant differences were found in age (*t *= 0.5600, *p *= 0.5776), gender (χ^2 ^= 1.763, *p *= 0.184), alcohol use (χ^2 ^= 0.287, *p *= 0.592), smoking habit (χ^2 ^= 2.052, *p *= 0.152) or tumor site (χ^2 ^= 2.400, *p *= 0.121) between the patients in the Tn group or the Tm/Lm groups (Table 2). As the translocation of p65 from the cytoplasm to the nucleus is required for NF-κB/relA signaling, the specimens were evaluated for nuclear p65 expression. As shown in Fig. 1a, p65 was markedly expressed in almost all of the SCCHN specimens. Positive nuclear staining was observed in 23% and 52% of primary tumors without lymph node involvement and primary tumors with lymph node involvement, respectively. Of the three groups, nuclear staining for p65 was most prevalent in lymph node contain metastases (61%) (Fig. 1b). The nuclear expression of NF-κB p65 was significantly different between the Tn, Tm and Lm groups (Wilcoxon rank-sum test; χ^2 ^= 37.489, *df *= 2, *p *= 0.001) (Fig. 1b). As half of the SCCHN samples were tongue squamous cell carcinoma, the statistic analysis was taken for squamous cell carcinoma in tongue and in other sites of head and neck individually. The similar results showed that the nuclear positive rates of NF-κB p65 in Lm and Tm groups were higher than Tn group (Wilcoxon rank-sum test; χ^2 ^= 14.573, *df *= 2, *p *= 0.001 in tongue group and χ^2 ^= 22.425, *df *= 2, *p *= 0.0001 in other site of head and neck group, respectively). These data demonstrate that NF-κB/relA activity can be correlated with SCCHN metastasis.

### Increased Constitutive NF-κB/relA Activity in Metastatic Human SCCHN Cell Lines and Increased Sensitivity to TNFα

**Figure 2:**
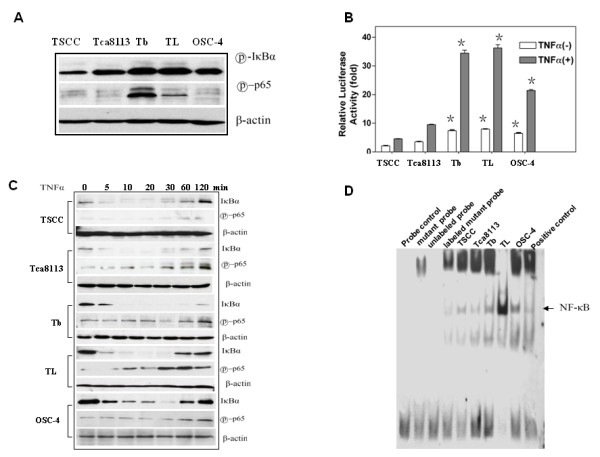
**Constitutive NF-κB activity in SCCHN cell lines**. (a) Western blot analysis of phosphorylated IκBα (serine 32) and phosphorylated NF-κB p65 (serine 536). (b) Constitutive NF-κB promoter activity. The asterisk (*) denotes *p *< 0.01 compared to untreated cells. The data shown are from one representative experiment out of three independent experiments. The bars indicate the standard deviation. (c) Western blot analysis of IκBα degradation and phosphorylated NF-κB p65 (serine 536) expression following treatment with TNFα for the indicated time. (d) NF-κB binding activity. Nuclear protein was extracted from HNSCC cells, and an EMSA was performed as described previously. The image is representative of three independent experiments.

To determine whether constitutive NF-κB/relA activity was present in SCCHN cells, we used a western blot to determine the level of phospho-IκBα in a number of SCCHN cells lines. The level of phospho-IκBα was high in the highly metastatic cell lines (Tb, TL and OSC-4), and phospho-p65 was highly expressed in the Tb and TL cell lines (Fig. 2a). Next, we co-transfected a 2× NF-κB-Luc reporter and a pPenilla Renilla reporter into these five SCCHN cell lines and assayed luciferase activity. As shown in Fig. 2b, the basal NF-κB promoter activity was significantly higher in the metastatic cell lines (Tb, TL, and OSC-4). When the cells were treated with TNFα, NF-κB promoter activity was further increased in the highly metastatic cell lines. Moreover, as detected using a western blot, IκBα was degraded more quickly in the Tb and TL cell lines. The constitutive NF-κB activities were significantly increased by the stimulation of TNFα in the Tb and TL cell lines (Fig. 2c). To confirm the high constitutive NF-κB activity in the highly metastatic cell lines, an EMSA was performed using nuclear extracts. While all of the five SCCHN cell lines showed NF-κB DNA-binding activity (Fig. 2d), the DNA binding was significantly higher in the highly metastatic cells lines (Tb, TL and OSC-4) compared to the cells with lower metastatic potential (TSCC and Tca8113).

### Down-regulation of Constitutive NF-κB/relA Activity stimulated by TNFα in Metastatic Human SCCHN Cells Treated with PDTC or BAY 11-7085

**Figure 3:**
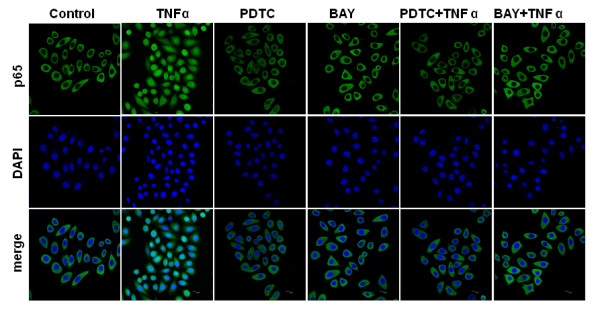
**Inhibition of NF-κB p65 nuclear localization by PDTC and BAY 11-7085**. The p65 expression in cells were observed using a Leica TCS SP2 confocal microscope. TNFα could promote the rapid nuclear localization of p65. Whereas, TNFα stimulation could not promote the translocation of p65 from the cytoplasm to the nucleus in PDTC- or BAY 11-7085-treated cells.

In order to use nuclear localization of p65 as a surrogate for NF-KB activity, the subcellular localization of NF-κB p65 was evaluated using immunofluorescence and confocal microscope. As shown in Fig. 3, the NF-κB activity was significantly higher in TL cells when treated with TNFα. TNFα promoted the rapid nuclear localization of p65. Whereas in PDTC- or BAY 11-7085-treated cells, TNFα stimulation could not promote the translocation of p65 from the cytoplasm to the nucleus.

### Suppression of Cell Migration in SCCHN Cells Treated with PDTC or BAY 11-7085

**Figure 4:**
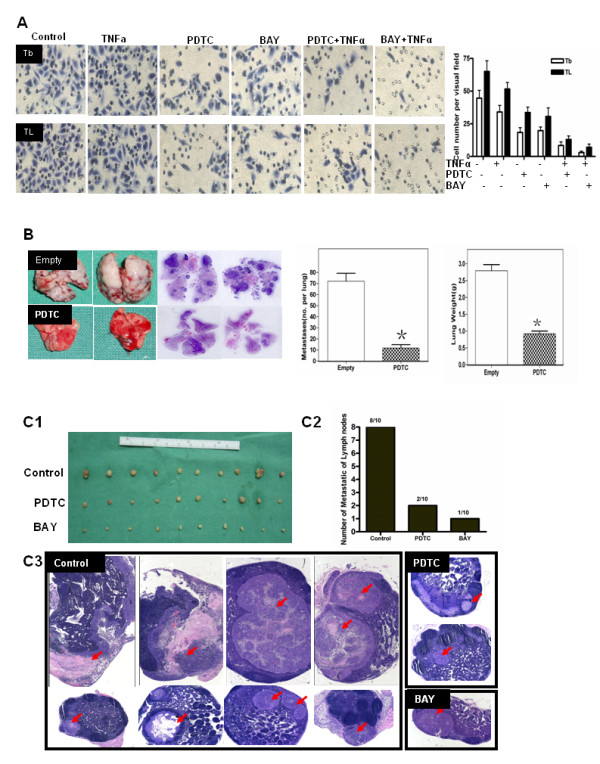
**Suppression of cell migration and metastasis in SCCHN cells following inhibition of the NF-κB signaling pathway**. (a) Treatment with either PDTC or BAY 11-7085 significantly decreased tumor cell invasion. TNFα stimulation further down-regulated cell invasion. (b) Decreased lung metastasis of Tb cells in nude mice. The weight of the lung was measured. The asterisk (*) indicates *p *< 0.01. (c) Decreased lymph node metastasis of TL cells in nude mice. The popliteal fossa lymph nodes were excised. (c1)Of 10 mice in each group, metastases were detected in eight lymph nodes in the control group, two in the PDTC group and one in the BAY 11-7085 group (c2). The popliteal fossa lymph nodes sections were observed using microscopy at 5× magnification (c3).

The effect of NF-κB activity on the migration of the highly metastatic SCCHN cell lines was evaluated using established an *in vitro *assay system. Tb and TL cell migration was inhibited 26.2% and 31.4%, respectively, when treated with PDTC and was inhibited 24.8% and 34.4%, respectively, when treated with BAY 11-7085 (Fig. 4a). Moreover, the migration of Tb cells was inhibited 36.2% and 41.6% when the cells were treated with either PDTC and TNFα or BAY 11-7085 and TNFα, respectively. TL cell migration was inhibited 52.0% and 58.0% following treatment with PDTC and TNFα or BAY 11-7085 and TNFα, respectively. Both PDTC and BAY 11-7085 produced a significant decrease in tumor cell migration (*p *= 0.001), and TNFα stimulation further decreased cell migration when the NF-κB pathway was inhibited (*p *< 0.05).

### Decreased Hematologic and lymphatic Metastasis of SCCHN Cell Lines Following Inhibition of NF-κB

To determine the effect of the NF-κB pathway on metastasis, Tb cells were injected into the lateral tail vein of mice, and these mice were treated with PDTC or with PBS (control) for two weeks. Six weeks after injection, both the PDTC-treated group and the control group developed metastases (Fig. 4b). Additionally, the Tb-PDTC group had significantly fewer (*p *= 0.0002) metastases (average number of 11.8 metastases per lung) than the control group (average number of 72.2 metastases per lung; Fig. 4b, left and middle panels). Additionally, the mice injected with Tb cells and treated with PDTC to block NF-κB activity showed a two-fold decrease in lung weight (Fig. 4b, right panel; *p *= 0.0002).

To further characterize the effect of NF-κB activity of metastasis, TL cells were injected into the hibateral foot pads of mice. The mice were either treated with PBS (control) or treated with PDTC or BAY 11-7085 for two weeks. Nine weeks after injection, the number of lymph nodes containing metastases was counted by three pathologists (Fig. 4c). In the control group, eight lymph nodes were positive for lymph node metastases, whereas two of the lymph nodes in the PDTC group had metastases, and one lymph node in the BAY 11-7085 group contained a metastasis (Fig. 4c2). These results demonstrate that decreased NF-κB activity leads to suppression of tumor metastasis.

### Cell Apoptosis Induced by PDTC or BAY 11-7085 and Sensitivity to TNFα

**Figure 5:**
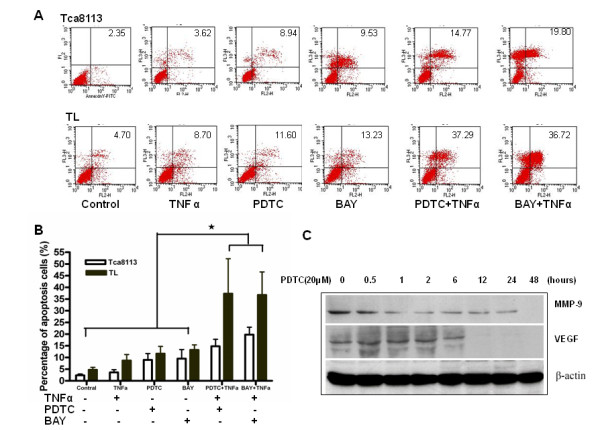
**Cell Apoptosis Induced by PDTC or BAY 11-7085 and Sensitivity to TNFα**. (a) Apoptosis induced by PDTC or BAY 11-7085 and sensitivity to TNFα. The data are from one representative experiment out of three independent experiments. (b) Cells treated with PDTC or BAY 11-7085 were more sensitive to TNFα-mediated killing; the asterisk (*) denotes *p *< 0.01. (c) Down-regulation of VEGF and MMP9 expression in PDTC-treated Tb cells.

To determine whether inhibition of NF-κB by PDTC or BAY 11-7085 increases the percentage of apoptotic cells and renders metastatic cancer cells sensitive to TNFα-mediated killing, apoptosis was measured using Annexin V staining following treatment with PDTC, BAY 11-7085 and/or TNFα (Fig. 5a). The rate of apoptosis in Tca8113 cells treated with PDTC or BAY 11-7085 reached to 6.59% and 7.18% from 2.62%, respectively, and the rate reached to 6.90% and 8.53% from 5.83% in TL cells treated with PDTC or BAY 11-7085, respectively (Fig. 5b). The percentage of apoptotic TL cells following treatment with PDTC or BAY 11-7085 was not significantly greater than either the control group or the TNFα treated group (*p *> 0.05). However, Tca8113 and TL cells treated with PDTC or BAY 11-7085 were more sensitive to TNFα-mediated killing (*p *= 0.0003) compared to the cells treated with TNFα alone. Significant differences in the apoptotic rate were found between the cells with lower metastatic potential (Tca8113) and the cells with higher metastatic potential (TL) after treatment with PDTC or BAY 11-7085 (*p *= 0.0120).

### Down-regulation of Metastasis-Associated Gene Expression in PDTC-treated Metastatic SCCHN Cells

Genes, such as vascular endothelial growth factor (VEGF) and matrix metalloproteinase-9 (MMP9), have been reported to be linked to tumor metastasis, and NF-κB binding sites have been identified in their promoters. Therefore, we examined whether PDTC treatment down-regulated VEGF and MMP9 expression in metastatic SCCHN cells. Tb cells were treated with PDTC for the indicated times and whole-cell extracts were prepared and analyzed using a western blot. PDTC treatment inhibited VEGF and MMP9 expression in a time-dependent manner (Fig. 5c). Similar results were found in TL cells treated with PDTC or BAY 11-7085 (data not shown).

## Discussion

The expression of p65 and IκB proteins in SCCHN specimens, normal mucosa and epithelial dysplasia specimens has been examined by immunohistochemistry. Several studies have suggested that the biological characters of SCCHN are correlated with the activity of NF-κB pathway [14,15]. Based on those findings, alterations in NF-κB activity may contribute to tumor invasion and metastasis *in vivo*. To confirm this possibility, we first assessed the activity of NF-κB pathway by observation of p65 nuclear localization in specimens from the patients with SCCHN. Our results show that the level of NF-κB activity differs significantly between primary tumors without lymph node involvement and primary tumors with lymph node involvement, as well as the metastatic lymph node. Furthermore, we have confirmed these clinical results through *in vitro *and *in vivo *experiments, suggesting that NF-κB activity contributes to SCCHN hematologic and lymphatic metastasis.

To study NF-κB activation and its role in SCCHN invasion and metastasis, we established the Tca8113 SCCHN cell line and generated the highly metastatic subclones, Tb and TL, through a combination of *in vivo *selection and *in vitro *cloning. Since the Tca8113, Tb and TL cell lines share an identical genetic background, we presumed that differentially expressed proteins and that different levels of signaling pathway activity play direct or indirect roles in invasion and metastasis. To solidify the data presented here, two additional SCCHN cell lines were added to the study, the poorly metastatic TSCC cell line and the strongly metastatic OSC-4 cell line. Using these five cell lines, we investigated the relationship between the level of NF-κB activity and SCCHN metastatic behaviour to explore the mechanism underlying SCCHN metastasis.

Our results show that the treatment of highly metastatic SCCHN cells with NF-κB inhibitors significantly reduced cell invasion *in vitro*, while the apoptotic rates in highly metastatic cells have not been significantly increased compared with the lower metastatic Tca8113 cells. Furthermore, NF-κB inhibitors significantly inhibited the metastasis of Tb cells to the lung and the metastasis of TL cells to the lymph node *in vivo*. These results demonstrate that inhibition of NF-κB/relA activity through suppression of IκBα phosphorylation and a reduction in the formation of active NF-κB is able to reduce tumor invasion and metastasis.

In our study, using both human cancer specimens and cell lines, clearly shows that NF-κB/relA activity significantly contributes to the metastasis of SCCHN. Although constitutive activation of NF-κB/Rel family members has been reported in several cancers [14-17], our data is the first time to demonstrate that NF-κB activity contributes to hematologic and lymphatic metastasis of SCCHN.

The mechanisms underlying high NF-κB/relA activity in SCCHN and other human cancer cell types are not clear. NF-κB has been reported to strongly regulate cell apoptosis in many tumor cells [18], which may be one of the possible mechanisms by which NF-κB inhibits cell invasion and metastasis. Our results show that NF-κB inhibitors could induce cell apoptosis. However, no significant difference in the apoptotic rate of cell lines with different metastatic potential was seen compared to the control groups. Therefore, there may be other mechanism contributing to NF-κB activity in SCCHN metastasis. Some studies have reported that the treatment of cancer cells with antisense RNA targeting p65 inhibited tumor cell adhesion and growth both *in vitro *and *in vivo *[19]. Studies have also demonstrated that NF-κB could regulate several proteins that promote tumor growth, invasion and metastasis, such as urokinase-type plasminogen activator and MMP9 [20,21]. In the present study, we found that proteins related to cell metastasis, including VEGF and MMP9, were down-regulated following inhibition of NF-κB, suggesting that these proteins may contribute to SCCHN metastasis. These data are consistent with previous studies examining other cancer cell lines.

Additionally, our results show that TNFα significantly induced the apoptosis of SCCHN cells treated with an NF-κB inhibitor. Recently, several studies have found that NF-κB functions as a tumor promoter in inflammation-associated cancer [6,22-24]. This finding may be the missing link between inflammation and cancer. As a result of this observation, many researchers have begun to focus on the interaction between the host environment and tumor cells. This tumor-host interaction could play a role in high level of NF-κB/RelA activity observed in malignant cells because the development of cancer metastases is determined by the interaction of tumor cells with their immediate environment, including tissue- or organ-specific cytokines [25]. A number of different stimuli can activate NF-κB. For example, inflammatory signals, hypoxia [26,27] and oncogenic proteins, such as mutated Ras [28], have been shown to regulate NF-κB activity. We observed that highly metastatic SCCHN cell lines constitutively produce proinflammatory cytokines, including TNFα, interleukin 1 α (IL-1α), IL-6, IL-8 and granulocyte macrophage-colony stimulating factor. Furthermore, the treatment of Tb or TL cells with PDTC or BAY 11-7085 inhibits the expression of these inflammatory cytokines (data not shown), which are known to activate the NF-κB pathway and to be induced in response to the activation of NF-κB/RelA-mediated transcription. Therefore, NF-κB activation, and a positive feedback loop, seems to play an important role in tumor metastasis. Additionally, NF-κB activation and interactions with the microenvironment most likely plays an important role not only in carcinogenesis, especially inflammation-associated cancers, but also in tumor invasion and metastasis.

Preliminary results have suggested that highly metastatic SCCHN cells are more sensitive to TNFα. Similarly, we found that the phosphorylation of p65 on serine 536 is increased in metastatic SCCHN cells. A number of recent studies have suggested that p65 phosphorylation may be necessary for transcriptionally competent nuclear NF-κB [29-32]. The lack of either of the kinases involved in NF-κB activation resulted in the normal activation of NF-κB in response to a wide variety of inducers, as measured by IκBα degradation, NF-κB nuclear translocation or binding to DNA; however, nuclear NF-κB was unable to drive transcription [33,34]. We hypothesize that the basal phosphorylation of p65 on serine 536 increased p65 transcription, thereby improving sensitivity to TNFα. Although Wang et al. reported that stimulation of cells with TNFα resulted in inducible p65 phosphorylation at position 529 [35], we detected increased phosphorylation of p65 on serine 536 when the cells were treated with TNFα. This result is consistent with a recent study that has suggested that TNFα can induce the phosphorylation of p65 on serine 536 [36]. In addition, several studies have shown that IL-1, lipopolysaccharide and phosphoinositide 3-kinase/Akt induced p65 phosphorylation on serine 536 [29,37-40], and overexpression of phosphorylated NF-κB were significant predictors of poor survival of tumor patients [41]. Therefore, we hypothesize that p65 phosphorylated on serine 536 may interact with additional signaling pathways that play an important role in SCCHN metastasis. Experiments testing this hypothesis are currently under investigation.

## Conclusions

Taking together, our previous and present studies demonstrate that NF-κB activity can be significantly correlated with SCCHN metastasis. The inhibition of the constitutive activity of the NF-κB signaling pathway reduces tumor hematologic and lymphatic metastasis through decreased expression of several proteins and cytokines related to metastasis. Therefore, the NF-κB pathway and its signaling effectors may be promising biomarkers to detect and treat SCCHN metastasis.

## Competing interests

The authors declare that they have no competing interests.

## Authors' contributions

WC and ZZ were responsible for the study design, interpretation of the data and revision of the manuscript. MY and PZ were responsible for data acquisition, analysis of the work presented and the preparation of the manuscript. MY, QX and XZ participated in the study of growth inhibition, cell apoptosis and determination of NF-kappa B activity. PZ and ZZ supervised the studies and helped to revise the manuscript. All authors read and approved the final manuscript.

## Pre-publication history

The pre-publication history for this paper can be accessed here:

http://www.biomedcentral.com/1471-2407/10/437/prepub
